# The Best Current Research on Patellar Tendinopathy: A Review of Published Meta-Analyses

**DOI:** 10.3390/sports12020046

**Published:** 2024-02-01

**Authors:** Rafael Llombart, Gonzalo Mariscal, Carlos Barrios, Rafael Llombart-Ais

**Affiliations:** 1 Orthopedic Surgery Department, University Clinic of Navarra, 31008 Pamplona, Spain; 2 Institute for Research on Musculoskeletal Disorders, School of Medicine, Valencia Catholic University, 46001 Valencia, Spain; 3 Traumacenter, Casa de Salud Hospital, 46021 Valencia, Spain

**Keywords:** patellar tendinopathy, sport, treatment, diagnosis, PRP, review

## Abstract

Patellar tendinopathy is a frequent overuse injury in sports that can cause significant pain and disability. It requires evidence-based guidelines on effective prevention and management. However, optimal treatments remain uncertain. We aimed to analyze available meta-analyses to summarize treatment recommendations, compare therapeutic modalities, examine included trials, and offer methodological suggestions to improve future systematic reviews. Meta-analyses were systematically searched for in PubMed (PROSPERO: CRD42023457963). A total of 21 meta-analyses were included. The AMSTAR-2 scale assessed study quality, which was low, with only 23.8% of the meta-analyses being of moderate quality, and none were considered to be of high quality. Heterogeneous outcomes are reported. Multiple platelet-rich plasma (PRP) injections appear superior to eccentric exercises and provide lasting improvements compared to eccentric exercises when conservative treatments fail. Extracorporeal shockwave therapy (ESWT) also seems superior to non-operative options and similar to surgery for patellar tendinopathy in the long term. However, evidence for eccentric exercise efficacy remains unclear due to inconclusive findings. Preliminary findings also emerged for genetic risk factors and diagnostic methods but require further confirmation. This review reveals a lack of high-quality evidence on optimal patellar tendinopathy treatments. While PRP and ESWT show promise, limitations persist. Further rigorous meta-analyses and trials are needed to strengthen the evidence base and guide clinical practice. Methodological enhancements are proposed to improve future meta-analyses.

## 1. Introduction

Patellar tendinopathy has gained relevance in recent years, especially in the sports field, constituting the second most frequent knee injury after medial collateral ligament injuries in athletes [1]. A prevalence of over 20% has been reported in collegiate basketball players, and 12% showed signs of patellar tendinopathy upon ultrasonography [2]. The incidence varies according to factors such as age, diagnostic methods, competitive level, and body morphotype [3,4], but there has been an increase in diagnosis due to the greater use of imaging techniques such as ultrasound and magnetic resonance imaging [5,6,7]. The use of these modalities has allowed for the identification of individuals at high risk of developing this condition, enabling the early detection of structural abnormalities to initiate unloading and treatment before the onset of symptoms, aiming to prevent progression to chronic conditions [8].

Patellar tendinopathy can have a significant impact on athletes, beyond its high prevalence rates. The condition is often referred to as “jumper’s knee” due to its frequent occurrence in jumping sports that involve repetitive knee extensor loading, such as basketball, volleyball, and track and field events. Athletes experiencing symptoms may struggle with pain, swelling, and stiffness in the knee during and after activity [9]. This can limit sporting performance and lead to the athlete having less time for training and competition [10]. In severe cases, patellar tendinopathy may result in an early retirement from competitive sports. The longevity of an athletic career is an important consideration, as prolonged symptoms and disability due to patellar tendinopathy disrupt the progression and achievement of athletic goals [11]. Furthermore, psychological factors such as fear-avoidance beliefs and reduced self-efficacy are common in athletes with chronic patellar tendon problems, which can compromise optimal sports participation [12].

Some biomechanical risk factors that have been investigated include hip range of motion and knee angle during landing, as well as quadricep flexibility [13]. However, despite this knowledge, prevention programs have not proven to be effective in reducing the risk and may even increase the likelihood of injury in individuals with preexisting tendon changes [14,15]. As a result, 10% of patients ultimately require surgery due to the failure of conservative treatments [16]. It is clear that more research is needed on preventive and therapeutic strategies for this prevalent condition.

There is controversy regarding the comparison of conservative treatments versus surgical interventions for patellar tendinopathy. Eccentric exercise has shown similar improvements to surgery in parameters such as The Victorian Institute of Sport Assessment Scale for Patellar Tendinopathy (VISA-P), strength, and pain, with no significant differences in satisfaction, return to sports, or overall assessment in the medium term [17]. However, surgical evidence may be biased by the way results are reported [17]. Among infiltrative therapies, corticosteroids provide short-term relief, while eccentric exercise is superior in the long term. Other options, such as polidocanol, aprotinin, and arthroscopic ablation, have shown promising preliminary results. Platelet-rich plasma (PRP) has been proposed as a second-line alternative for refractory tendinopathies, although its use needs to be optimized based on PRP biology and application modalities [18]. Recent evidence suggests that PRP has a greater benefit compared to extracorporeal shockwave therapy, but more randomized trials are needed before recommending it as the standard over non-PRP injections [19,20]. Despite advancements such as shockwave therapy or cryotherapy, surgery is still necessary in advanced cases refractory to previous conservative treatments. Open approaches such as debridement are technically straightforward and effective in the long term [6]. Arthroscopic techniques are gaining relevance due to their advantages [21,22].

The main objective of this review is to analyze the available meta-analyses on the treatment of patellar tendinopathy to summarize recommendations based on the best current evidence. Additionally, we aimed to examine the characteristics and results of the included clinical trials, comparing the effectiveness between therapeutic modalities and identifying areas that require further high-quality research on this condition in the future. Finally, we aimed to provide methodological recommendations to improve the quality of systematic reviews and meta-analyses in this field.

## 2. Materials and Methods

### 2.1. Eligibility Criteria

This study had a written protocol and was registered in PROSPERO (CRD42023457963). The study followed the Preferred Reporting Items for Systematic Reviews and Meta-Analyses (PRISMA) guidelines [23]. The research question for this study focused on patellar tendinopathy. Specifically, this meta-analysis aimed to review the available meta-analyses on patellar tendinopathy. No particular intervention or comparison group was specified. The outcomes of interest included risk factors, conservative treatment approaches, injectable treatments, surgical interventions, early diagnosis methods, prevention strategies, and other related topics. The included studies consisted of meta-analyses that examined patellar tendinopathy. The exclusion criteria were studies that did not include any research related to patellar tendinopathy, duplicate studies, studies that were not meta-analyses (such as narrative reviews), and other irrelevant publications.

### 2.2. Information Sources and Search Methods for Identifying Studies

The search was conducted in PubMed. No language or date restrictions were applied. The search term used was (“patellar tendinopathy”) with a filter of meta-analysis. The search was conducted by two independent reviewers who screened the studies for eligibility. Any disagreements were resolved through discussion, and a consensus was reached on which studies to include.

### 2.3. Data Extraction and Data Items

Regarding data extraction, two authors independently reviewed the studies. In the event of disagreement, a third review author was consulted to achieve a consensus. From each study, the following variables were obtained: study, region, journal, pathology, protocol, population, n studies, diagnosis method, compared treatments, main outcomes, risk of bias or quality, limitations, and main conclusions.

### 2.4. Assessment of the Risk of Bias in the Included Studies

For the assessment of the quality of the previously published meta-analyses, we extracted the variables required by the AMSTAR-2 scale. AMSTAR-2 is a tool that allows for a detailed assessment of meta-analyses and systematic reviews of RCTs and non-randomized studies. AMSTAR-2 is a questionnaire with 16 domains that requires simple answers: yes (positive result), no (insufficient information), or partial yes (partial information to standard) [24].

### 2.5. Assessment of Results

To carry out our evaluation of the results described in the studies included in this review, an Excel spreadsheet was used, in which the most relevant data from each article were systematically collected. After completing data collection using the spreadsheet, a detailed narrative review that allowed for the main findings described in the different studies to be integrated and synthesized was carried out. Finally, a summary table was created to graphically show and compare the most relevant results.

## 3. Results

### 3.1. Study Characteristics

A systematic search of PubMed for meta-analyses on patellar tendinopathy was performed, leading to the identification of a total of 21 studies that met the inclusion criteria [14,25,26,27,28,29,30,31,32,33,34,35,36,37,38,39,40,41,42,43,44] (Table 1). Regarding the origin of the meta-analyses, three were from the United States, two were from Italy, one was from New Zealand, three were from the United Kingdom, three were from Australia, one was from China, one was from Ireland, one was from Canada, one was from the Netherlands, one was from Brazil, one was from Spain, one was from Finland, and one was from Belgium. This reflects that research on tendinopathies through systematic reviews and meta-analyses has been approached by groups from diverse regions, mainly Europe, North America, and Australasia. Regarding the type of publishing journal, 15 meta-analyses were in sports medicine journals, 2 were in the fields of rehabilitation and physical therapy, and the remainder were in journals of various topics. This was expected, given that tendinopathies are injuries frequently associated with sports practice. Regarding the anatomical location, six studies focused exclusively on patellar tendinopathy, three included both Achilles and patellar tendinopathy, and the rest addressed lower-limb tendinopathy or knee tendinopathy more generally. Only a few of the meta-analyses specifically analyzed patellar tendinopathy. A concerning finding was that only 6 of the 21 meta-analyses had a previously registered protocol (i.e., PROSPERO). This database allows for the registration of systematic review protocols, promoting transparency and methodological quality. The lack of a preregistered protocol can bias results by enabling post hoc changes to eligibility criteria and analysis. Regarding the type of population, most meta-analyses did not delimit the sample to a specific subgroup. The few that did focus the population centered on tendinopathy in athletes. The number of primary studies included in the meta-analyses varied widely between 11 and 52 articles. Specifically those on patellar tendinopathy included between 1 and 28 studies. Heterogeneity was observed between the reviews, with some including primarily high-quality studies and others with a significant proportion of medium- or low-quality studies.

### 3.2. AMSTAR-2

Regarding the quality of the meta-analyses (Table 2), 10/21 (47.6%) presented critically low-quality studies, 6/21 (28.6%) presented low-quality studies, and 5/21 (23.8%) presented moderate-quality studies. There were no studies of high or very high quality. Only 7/21 (33.3%) studies strictly followed the PICO(S) search strategy. Overall, 10/21 (47.6%) presented a registered protocol. The inclusion and exclusion criteria were adequately detailed, and study selection and data extraction were carried out in duplicate in most cases. Almost all used appropriate tools for assessing the risk of bias. Overall, 11/21 (52.4%) provided an adequate assessment of publication bias. Finally, all studies failed to report the influence the funding the author(s) received or conflicts of interest had on the results.

1. Did the research questions and inclusion criteria for the review include the components of PICO?
2. Did the report of the review contain an explicit statement that the review methods were established prior to the conduction of the review, and did the report justify any significant deviations from the protocol?
3. Did the review authors explain their selection of the studies included in the review?
4. Did the review authors use a comprehensive literature search strategy?
5. Did the review authors perform study selection in duplicate?
6. Did the review authors perform data extraction in duplicate?
7. Did the review authors provide a list of excluded studies and justify their exclusions?
8. Did the review authors describe the included studies in adequate detail?
9. Did the review authors use a satisfactory technique for assessing the risk of bias (RoB) in the individual studies that were included in the review?
10. Did the review authors report on the sources of funding for the studies included in the review?
11. If a meta-analysis was performed, did the review authors use appropriate methods for statistically combining results?
12. If a meta-analysis was performed, did the review authors assess the potential impact of the RoB in individual studies on the results of the meta-analysis or other evidence synthesis?
13. Did the review authors account for the RoB in individual studies when interpreting/discussing the results of the review?
14. Did the review authors provide a satisfactory explanation for, and discussion of, any heterogeneity observed in the results of the review?
15. If they performed quantitative synthesis, did the review authors carry out an adequate investigation of publication bias (small study bias) and discuss its likely impact on the results of the review?
16. Did the review authors report any potential sources of conflicts of interest, including any funding they received for conducting the review?

### 3.3. Summary of Main Outcomes (Table 3 and Table 4)

#### Risk Factors Associated with Patellar Tendinopathy

Several meta-analyses have examined the risk factors associated with patellar tendinopathy and have found contradictory evidence regarding their influence on the development of this condition. Some of the factors studied include ankle dorsiflexion range of motion, hamstring and quadricep flexibility, jump training volume, countermovement jump (CMJ) height, and activity volume [42].

**Table 3:** The main results, limitations, and conclusions of the included meta-analyses that focused on comparative treatments.

Study	Main Outcomes	Limitations	Conclusions
Andriolo et al., 2018 [25]	VISA-P.	Different exercise protocols.	Positive overall outcome for treatments of patellar tendinopathy. Multiple PRP injections show potential as a treatment that is superior to eccentric exercises and lasting results compared to eccentric exercises, particularly in cases where conservative approaches have proven ineffective.
Woodley et al., 2007 [44]	IASP, VAS, McGill Pain Questionnaire, Function.	NS.	The effectiveness of eccentric exercise therapy for common tendinopathies remains unclear due to a lack of high-quality research, along with inconclusive results. Further rigorous trials are necessary to determine its optimal dose–response and long-term efficacy compared to other treatments.
Mani-Babu et al., 2014 [32]	VAS, Harris Hip Score, Roles and Maudsley Score, Mid-Portion.	NS.	For PT, ESWT seems to be superior to other non-operative treatments and equal to surgery in the long term.
Wang et al., 2023 [14]	Frequency or incidence of PT.	NS.	The risk of PT cannot be reduced with the current prophylactic program. However, for athletes, the negative results may be due to an insufficient sample size.
Masiello et al., 2023 [33]	VAS, VISA-P.	NS.	The limited available evidence shows no significant difference between PRP and control groups for pain or function in patellar tendinopathy.
Khan et al., 2023 [30]	Pain, functional.	Variability in both the number and dosage of the HA.	The single study on PRP versus HA for proximal patellar tendinopathy found that PRP was superior for improving pain and quadricep strength.
Chen et al., 2019 [26]	Pain.	No functional outcomes.	PRP may provide both short-term and long-term pain relief for tendon and ligament injuries and pathologies compared to alternative treatments.
Moraes et al., 2014 [36]	Disabilities of the Arm, Shoulder, and Hand questionnaire (Hudak 1996), Victorian Institute of Sports Assessment—Achilles questionnaire (VISA-A), and American Orthopaedic Foot and Ankle Society (AOFAS) foot questionnaire. Visual analogue scales (VASs) and local and systemic adverse effects.	Some studies may have been missed since PRP is an emerging field; extensive contacts were made with experts to find unpublished data.	The current evidence is insufficient to support the use of PRP therapy for treating musculoskeletal soft tissue injuries, both overall and for specific clinical conditions. When considering future RCTs, researchers should evaluate ongoing trials to determine the need for additional studies on particular injuries. The standardization of PRP preparation protocols is warranted.
Saltychev et al., 2022 [40]	Pain: VAS or NRS.	Great heterogeneity.	There was no evidence that NTG is more effective in reducing pain in Achilles tendinopathies, as well as patellar tendinopathies.
Shim et al., 2023 [40]	Global Rating of Change (GROC), VAS.	NS.	Patients report general satisfaction and positive perceptions of exercise therapy for tendinopathy, but more focus on these outcomes is recommended.

ESWT: extracorporeal Shockwave therapy; HA: hyaluronic acid; IASP: International Association for the Study of Pain; NRS: numeric rating scale; NS: not specified; NTG: nitroglycerin; PRP: platelet-rich plasma; PT: patellar tendinopathy; VAS: visual analogue scale; VISA-P: Victorian Institute of Sport Assessment scale for patellar tendinopathy.

**Table 4:** The main findings, limitations, and conclusions of the included meta-analyses that focused on risk factors, diagnosis, and miscellaneous factors.

Study	Main Outcomes	Limitations	Conclusions
Sprague et al., 2018 [42]	CMJ height, standing jump height, knee extension torque, knee flexion torque, occupational classification, knee loading during work.	Few women and non-athletes; different definitions of tendinitis.	Conflicting evidence that decreased ankle dorsiflexion range of motion, decreased posterior thigh and quadricep flexibility, greater volume of jump training, more volleyball sets played per week, greater CMJ height, and greater activity volume are potential modifiable risk factors.
Tayfur et al., 2022 [43]	Kinematic variables: initial contact angles of joints (hip, knee, ankle) or segments (i.e., trunk); range of motion (RoM) and peak angles in the same joints or segments; and joint angular velocities. Kinetic variables such as joint moments, peak ground reaction forces (GRF) in both horizontal and vertical planes, peak patellar tendon force (PTF), and lower-limb muscle activation patterns.	Few women; heterogeneity in tasks implemented, populations, and variables.	Three recommendations: improve ankle dorsiflexion–plantarflexion range, optimize trunk flexion strategies, and use soft landing patterns.
Obst et al., 2018 [38]	VISA-P, duration of symptoms, strain and stiffness, modulus, knee extension contractions using 2D ultrasound combined with dynamometry.	No quantitative 3D ultrasound.	Insufficient evidence exists to establish a consistent impact of tendinopathy on the mechanical properties of the patellar tendon, suggesting that the location and nature of the pathology may not significantly affect its overall tensile behavior in vivo.
McAuliffe et al., 2016 [35]	US and symptoms.	Reliability, variability in terms of terminology used in defining what is accepted as a structurally ‘abnormal’ tendon; lack of gold-standard tests for diagnosing tendinopathy.	Tendon abnormalities are predictive of the development of future Achilles or patellar tendinopathies.
Mousavi et al., 2019 [37]	Kinematics: running mileage, speed, gait phase, diagnosis, gait analysis tools and testing conditions.	Great variety of diagnostic methods.	Kinematic analysis showed that patellar tendinopathy patients had increased peak ankle eversion, hip adduction, and decreased tibial rotation compared to the controls. No other differences were significant.
Kim et al., 2021 [31]	Gene.	NS.	Genetic markers in COA1 seem to be associated with patellar tendinopathy and are potential risk factors for patellar tendinopathy that deserve further validation regarding molecular mechanisms.
Palazón-Bru et al., 2021 [39]	Reliability VISA-P.	NS.	The reliability of VISA-P for assessing the severity of patellar tendinopathies requires greater evaluation with more scientific evidence before it can be implemented in clinical practice.
Heales et al., 2013 [29]	Pain, sensory and motor function.	Lack of confirmatory diagnosis with image (just two studies).	Tendinopathy patients frequently have sensory and motor deficits in their non-injured limb, indicating CNS involvement beyond just local pathology. The contralateral side should not be used as a reference in assessment.
Matthews et al., 2018 [34]	Endonmatrix changes (e.g., tendon thickness, echogenicity, collagen organization, fibrillar pattern, vascularization.	NS.	US-based tendinopathy diagnosis criteria are variable. Ultrasound predicts future symptoms. Assessing tendon structure with three parameters has a higher predictive value than using two parameters.
De oliveira Silva et al., 2019 [28]	Remote pressure pain thresholds, remote thermal pain thresholds, pain assessments of manifestations of pain sensitization.	Need for multivariate analysis.	The findings in people with patellar tendinopathy were conflicting.
De Bleecker et al., 2020 [27]	Kinematics: type of jump, phase of landing, plane of movement, and main results.	NS.	Preliminary evidence links impaired local and non-local landing kinematics with lower-extremity overuse injuries. Excess frontal and transverse plane motions during jumping/landing may increase forces on lower-limb structures.

CNS: central nervous system; CMJ: countermovement jump; NS: not specified; PT: patellar tendinopathy; US: ultrasound; VISA-P: Victorian Institute of Sport Assessment scale for patellar tendinopathy.

Ankle dorsiflexion range of motion and hamstring and quadricep flexibility have been identified as potential risk factors for patellar tendinopathy. However, the results of meta-analyses have been contradictory regarding their influence. Some studies suggest that a limited dorsiflexion range of motion and reduced flexibility in the posterior thigh muscles and quadriceps may increase the risk of developing this condition. On the other hand, other studies have not found a significant association between these factors and patellar tendinopathy. These discrepancies could be due to differences in the populations studied, measurement methods used, and other confounding factors [42].

Improving ankle dorsiflexion–plantarflexion range of motion has been suggested to be beneficial in reducing the risk of patellar tendinopathy. This can be achieved through the implementation of specific ankle stretching and mobility exercise programs. These exercises can include calf stretches, joint mobilization movements, and foot and leg muscle strengthening exercises. By improving ankle flexibility and range of motion, load and tension on the patellar tendon during physical activity can be reduced [43].

The optimization of trunk flexion strategies during physical activity may also be important for reducing the risk of patellar tendinopathy. Certain trunk motion patterns, such as greater forward leaning or excessive flexion, have been observed to increase load on the patellar tendon [43]. Therefore, it is advisable to work on core muscle strengthening and improve movement techniques to maintain proper posture and adequate trunk alignment during physical activity [43].

Jump training volume, especially in sports like volleyball, and the number of volleyball sets played per week have also been investigated as potential risk factors for patellar tendinopathy. As with the previous factors, the results are inconsistent. Some studies have found a positive association between greater jump training volume and a higher risk of developing patellar tendinopathy. However, other studies have not found a significant relationship. These discrepancies could be attributed to variability in training methods, activity intensity, and individual athlete characteristics [42,43].

CMJ height and physical activity volume have also been evaluated as risk factors for patellar tendinopathy. As in previous cases, the results are contradictory. Some studies suggest that higher CMJ height and greater physical activity volume can increase the risk of developing this condition. However, other studies have not found a significant association. These discrepancies could be due to the lack of standardization in measuring CMJ height, as well as differences in individual participant characteristics [42].

Soft and controlled landing patterns are also important for reducing the risk of patellar tendinopathy. Excessive movements in the frontal and transverse planes during jumps and landings have been observed to increase forces on lower-extremity structures, including the patellar tendon. It is advisable to learn and practice soft, controlled landing techniques involving proper impact absorption and efficient force distribution along musculoskeletal structures [27].

It is important to consider that these risk factors may interact with each other and with other individual factors such as genetics, age, and general fitness level. Additional research is needed to better understand the relationship between these factors and patellar tendinopathy, which will allow for more effective prevention and treatment strategies to be developed.

### 3.4. Platelet-Rich Plasma

Platelet-rich plasma (PRP) injection therapy has gained popularity as a treatment option for patellar tendinopathy. In general, the results of these studies have demonstrated an overall positive outcome for treating patellar tendinopathy with PRP injections. It has been observed that multiple PRP injections show superior potential and lasting results compared to eccentric exercises, especially in cases where conservative approaches have been ineffective [25,26].

However, it is important to note that the available evidence is limited and that results may vary between studies. Some studies have found no significant difference in terms of pain or function between PRP-treated groups and control groups in patellar tendinopathy [33]. These findings may indicate that PRP does not provide additional benefits in terms of pain relief or functional improvement compared to conventional treatments used in control groups.

Despite discrepancies in results, one specific study comparing PRP to hyaluronic acid (HA) for proximal patellar tendinopathy found that PRP was superior for improving pain and quadricep strength [30].

It is important to highlight that while PRP has shown potential benefits in treating patellar tendinopathy, there are still several unanswered questions and areas of uncertainty regarding its use. For example, the optimal PRP dose and the frequency and timing of injections, as well as proper patient selection for those who would most benefit from this treatment, have not been fully established yet [36]. More research and well-designed clinical trials are needed to address these gaps in knowledge and provide a solid foundation to support the use of PRP in treating patellar tendinopathy.

Additionally, it is important to note that PRP is not the only available treatment option for patellar tendinopathy. Other conservative approaches such as eccentric exercises, physiotherapy, anti-inflammatory medication, and modifications in physical activity are also widely used in managing this condition. Choosing the most appropriate treatment for a particular patient should be based on a full assessment of their condition, including symptom severity, response to previous treatments, and individual patient preferences.

### 3.5. Physical Therapies or Rehabilitation

The potential benefits of eccentric exercise therapy in treating common tendinopathies remains a topic of ongoing debate, largely due to an absence of high-quality, conclusive research [41]. Eccentric exercises, characterized by the controlled lengthening of a muscle under tension, are theorized to strengthen affected tendons and encourage healing. Despite the extensive research in this area, a clear consensus on its efficacy relative to other tendinopathy treatments is still to be reached. The paucity of definitive research poses a significant roadblock to determining the value of eccentric exercise therapy. To remedy this, more rigorous clinical trials are needed to evaluate the optimal dosage, response, and long-term efficacy of this therapy compared to other interventions [44].

Preventive measures such as prophylactic programs have also been implemented to mitigate injury risk in athletes and other vulnerable individuals. However, the effectiveness of these programs in preventing tendinopathies remains inconclusive. In particular, the results of the patellar tendinopathy prophylactic program, in terms of reducing injury risk, have been disappointing. It is important to acknowledge that these outcomes may be skewed by the insufficient sample sizes in athlete studies, which may impede the discernment of significant differences and the drawing of definitive conclusions about the prophylactic program’s effectiveness [14,40].

### 3.6. Extracorporeal Shockwave Therapy

Extracorporeal shockwave therapy (ESWT) has demonstrated potential in reducing pain and enhancing functional improvement compared to placebo or other non-surgical therapies including NSAIDs, physical therapy, and injections [32]. Theories suggest that the beneficial effects of ESWT may be attributed to stimulated angiogenesis, augmented vascularization, and the regeneration of damaged tendon tissue [32]. Further research is needed to comprehend the physiological mechanisms underlying these potential benefits.

### 3.7. Ultrasound

Ultrasound technology has gained recognition as a promising tool for predicting and evaluating the progression of tendinopathies, especially in Achilles and patellar tendons. Evidence from several longitudinal studies indicates that ultrasounds can identify early structural abnormalities in tendons, which may be helpful in predicting the subsequent manifestation of clinical symptoms and dysfunction [34,35]. Despite its predictive utility, ultrasound-based diagnosis is hampered by methodological limitations that complicate comparisons between studies and the standardization of diagnostic criteria. Given the absence of a consensus on a standardized protocol for ultrasound-based evaluations of tendinopathies, further research is needed to address these limitations and improve diagnostic accuracy.

### 3.8. Miscellaneous

#### 3.8.1. Genetics

Recent investigations have identified potential genetic markers in the COA1 gene that may be associated with patellar tendinopathy, suggesting a potential genetic predisposition to this condition. Despite these preliminary findings, evidence on the genetic underpinnings of tendinopathies remains limited, and the field requires further exploration to identify reproducible genetic markers linked to these conditions [31].

#### 3.8.2. Reliability

The VISA-P questionnaire, designed to assess the severity of patellar tendinopathy, is a promising tool. However, its reliability requires further validation through larger, methodologically robust studies to determine its test–retest reliability, internal consistency, construct validity, and ability to detect clinical changes. Despite the current lack of sufficient evidence to recommend VISA-P’s routine use in clinical practice, it shows promise as a standardized tool for research and follow-up [39].

#### 3.8.3. Central Sensitization

There is currently insufficient evidence to comprehensively establish the impact of tendinopathy on the mechanical properties of the patellar tendon [38]. Moreover, patients with tendinopathies often exhibit sensory and motor deficits in their uninjured extremities, suggesting the broader involvement of the central nervous system [28]. Thus, the contralateral side should not be used as a reference in assessments [29]. Studies on central sensitization in patients with patellar tendinopathy have yielded contradictory findings, preventing the establishment of a clear association [28,29].

#### 3.8.4. Nitroglycerin

Topical nitroglycerin (NTG), commonly used to treat angina by dilating blood vessels and increasing blood flow, has been explored as a potential treatment for Achilles and patellar tendinopathies. However, the current literature lacks evidence supporting the efficacy of NTG in reducing pain associated with these conditions. This absence of evidence does not necessarily indicate ineffectiveness but rather emphasizes the need for high-quality studies to evaluate the potential use of NTG in the context of Achilles and patellar tendinopathies [40].

## 4. Discussion

Patellar tendinopathy is a condition that has been researched more frequently in recent years, as evidenced by a greater number of articles in specialized journals. In this meta-analysis review, it was observed that 14 of the 21 included studies were published in sports medicine journals, and 2 of the 21 included studies were published in rehabilitation and physical therapy journals, which could reflect potential differences in how patellar tendinopathy is approached. However, despite growing interest, the quality of the available evidence still presents important limitations. Only 6 of the 21 meta-analyses focused exclusively on patellar pathology, while most included various anatomical locations, generating heterogeneity and hindering specific conclusions. Additionally, the lack of preregistered protocols, present in most reviews, compromises methodological quality according to criteria such as the AMSTAR-2 scale. While the number of primary studies included in the meta-analyses was adequate in several cases, the number of studies specifically on patellar tendinopathy was very low, reaching only 1 or 2 studies in some reviews. Follow-up times were often insufficient to evaluate long-term effects. The quality of primary studies varied widely depending on design, and there persists a tendency to use clinical criteria for diagnosis as an inclusion criterion rather than more objective imaging methods.

First, eccentric exercise has been shown to be beneficial for treating patellar tendinopathy [45]. However, there is some controversy around its effectiveness due to the differences in the exercise regimens used in various studies. There is evidence suggesting that eccentric exercise can improve the short-term symptoms of patellar tendinopathy [25]. However, it may be difficult to determine if these exercises have been performed adequately and if exercise regimens are sufficient. Additionally, there may not be a “one size fits all” approach in terms of exercise regimens, and it may be necessary to customize exercises according to individual patient needs.

Extracorporeal shockwave therapy (ESWT) and platelet-rich plasma (PRP) therapy have been proposed as potential treatments for patellar tendinopathy. Both have a physiological basis for use in treating tendon disorders and have shown promising results in some studies [32,46]. However, study-derived results on PRP have been heterogeneous, and while it may have a greater effect in conditions like epicondylitis, this effectiveness has not been consistently demonstrated in patellar tendinopathy [26]. This may be due, in part, to differences in the composition of the PRPs used in studies and differences between soft tissues in general, including the differences between tendons and ligaments [47].

It has been suggested that multiple PRP injections may be needed to maintain their long-term beneficial effect [25]. This is an area that requires more research to determine the optimal relationship between the number of injections and clinical benefits.

Movement kinematics, particularly landing, have also been understudied in relation to patellar tendinopathy. However, there is evidence suggesting that excessive movements in the frontal and transverse planes can increase loads on the patellar tendon and increase injury risk [27]. Two-dimensional video analysis can be a useful tool for detecting altered landing patterns and may have value in patellar tendinopathy prevention and rehabilitation [48].

In terms of patient pain response, patients with patellar tendinopathy have been observed to have greater central sensitization, with allodynia and lower pain thresholds [49]. However, exercise has also been observed to reduce hyperalgesia in these patients [28]. This underscores the importance of exercise as part of patellar tendinopathy management, not just to improve tendon function but also to manage pain symptoms.

Ultrasound has demonstrated its value in predicting future tendon injuries or symptoms, based mainly on tendon echogenicity and thickness. Some of these ultrasonographic anomalies include hyperechoic zones, thin thickness, and neovascularization [34]. This may be especially relevant in elite athletes participating in jumping sports due to the high demand placed on their patellar tendon and incidence of patellar tendinopathy in this population [3,4]. However, prevention program results have been controversial [14].

Additionally, there are important considerations when using ultrasound for patellar tendinopathy diagnosis and follow-up. On the one hand, there is the possibility of finding anomalies in healthy people, which can lead to overdiagnosis. Subjectivity in ultrasound image interpretation is also inherent.

Additionally, sociodemographic, psychological, lifestyle, and social factors can influence tendon status assessment [35]. For example, psychosocial issues such as sleep, rest disruptions, fatigue, and anxiety can influence symptom perception and disease management [35]. This underscores the importance of a holistic approach to patellar tendinopathy management, focusing not just on the tendon itself but on the whole individual.

Patient-specific factors can also play a role in developing patellar tendinopathy. For example, hip adduction and an imbalance between internal and external tibial rotation, along with foot/ankle eversion, have been identified as risk factors for patellar tendinopathy [37]. Furthermore, quadricep flexibility and strength are essential for patellar tendon health [42]. Decreased hip extension has also been identified as a risk factor, possibly related to stiffness during landing [42].

In general, patellar tendinopathy is a multifactorial condition requiring a multifaceted treatment approach. Exercise, both eccentric and of other types, can play a valuable role in managing the condition. Extracorporeal shockwave therapy and platelet-rich plasma therapies may provide benefits, but more research is needed to define their roles. Ultrasound technology can be useful for diagnosis and monitoring, but it should be used cautiously due to the possibility of overdiagnosis and the potential psychological effects of an anomaly diagnosis. Finally, intrinsic patient factors and lifestyle factors must be considered when managing patellar tendinopathy.

### Limitations

This review presents some limitations, such as the exclusive inclusion of meta-analyses and not narrative reviews, the combination of different anatomical locations of tendinopathy in several studies, and the presence of heterogeneity from mixing separate populations such as athletes and non-athletes. Another limitation of this review was that the inclusion criteria may have been too broad. Studies were eligible for inclusion if they involved patellar tendinopathy, and no restrictions on the specific sport or competition level were implemented. However, the manifestation and impact of patellar tendinopathy could vary substantially between recreational and elite professional athletes or sports with jumping versus sports without jumping. The elimination of duplicate or outdated versions of the same meta-analyses may have biased our results. Similarly, by not globally presenting the total sample size, follow-up time, mean age, and number of women included in each review, it is difficult to have an overview of the demographic characteristics. In addition, the lack of stratification based on the quantitative tendon abnormality assessment in the current study is a limitation. Not accounting for the degree or severity of the underlying pathology could certainly contribute to heterogeneity between individuals and insufficiently conclusive findings. Future research should aim to directly correlate tendinopathy treatment outcomes with objectively graded tendon status, as determined through imaging modalities such as ultrasound or MRI. Stratifying analyses and comparisons according to mild, moderate, or severe tendon changes could help determine whether particular interventions are preferentially effective for certain phenotypes. This targeted approach, based on the degree of structural abnormality, has the potential to better elucidate how treatment responses may vary depending on the baseline tendon status. Prospective studies quantifying the tendon status both before and after planned therapies could also provide novel insights.

However, this review’s strengths include the use of validated scales to evaluate pain and functionality and the large sample sizes in the primary studies included within the meta-analyses.

## 5. Conclusions

This systematic review highlights the variations in findings and the need for additional high-quality research in key areas related to patellar tendinopathy. While certain risk factors, such as decreased ankle dorsiflexion, show conflicting evidence, modifiable ones, including flexibility imbalances and high training volumes, warrant targeted interventions. Based on these results, clinicians should consider multiple PRP injections over eccentric exercises alone for refractory cases while optimizing PRP protocols. ESWT has emerged as an option that is superior to non-surgical alternatives and comparable to long-term surgery, indicating that it may be particularly effective for patients failing conservative management. To increase the efficacy of eccentric exercise, future studies should establish ideal dosing and long-term comparative outcomes with other options. Objective assessment tools such as ultrasound demonstrate potential utility, necessitating standardized diagnostic criteria. Certain genetic markers appear to be linked to patellar tendinopathy risk but require further mechanistic validation. Personalized care considers each patient’s presentation, comorbidities, and aims.

To strengthen the evidence base and address the current limitations, rigorously designed primary research studies are needed. Future studies should employ standardized diagnostic criteria, exercise protocols, and clearly defined tendinopathy classifications to facilitate comparisons across studies. Incorporating quantitative 3D ultrasonography and establishing the reliability of diagnostic approaches could provide a more objective evaluation. Additionally, stratified analyses of PRP protocols and standardized HA dosing variables are warranted to clarify the optimal interventions. Utilizing larger and more diverse sample populations that include more females and non-athletes would enhance generalizability. The consistent use of validated functional outcomes beyond pain, as well as the confirmation of tendinopathy diagnoses with imaging modalities, is also important to consider. Researchers should further aim to reduce heterogeneity through homogeneous sampling, clearly reported methodologies, and multivariate statistical analyses that account for potential confounding factors. Facilitating comparisons via well-designed trials with standardized methodologies could help clarify inconsistencies, evaluate new treatments, and definitively address existing gaps in tendinopathy evaluation and management.

## Figures and Tables

**Table 1:** Baseline characteristics of the 21 included meta-analysis.

Study	Region	Journal	Pathology	PROTOCOL	Population	n Studies	Diagnosis Method	Compared Treatment *
Andriolo et al., 2018 [25]	Italy	Am J Sports Med	Patellar tendinopathy	NS	NSP	22	NS	Non-surgical treatments
Chen et al., 2019 [26]	USA	Am J Sports Med.	Tendons and ligaments	NS	NSP	21 (2 patellar)	Clinically	PRP vs. others
De Bleecker et al., 2020 [27]	Belgium	Sports Med	Lower-extremity overuse injuries	CRD42019135602	Physically active populations	12 (9 patellar)	NS	NA
De oliveira Silva et al., 2019 [28]	Brazil	Pain Med	Painful knee disorders	CRD42015024211	Knee joint in people with painful knee disorders	52 (3 patellar)	Manifestations of pain sensitization measured locally	NA
Heales et al., 2013 [29]	Australia	Br J Sports Med	Bilateral measurement in patients with unilateral tendinopathy	NS	Bilateral measurements in patients with unilateral tendinopathy	20 (1 patellar)	Clinical	NA
Khan et al., 2023 [30]	Canada	Sports Health	Soft tissue	NS	NSP	19 (1 patellar)	NS	HA vs. PRP
Kim et al., 2021 [31]	USA	Med Sci Sports Exerc	Patellar tendonitis	NS	NSP	NA	Gene	NA
Mani-Babu et al., 2014 [32]	UK	Am J Sports Med	Lower-limb tendinopathy	NS	NSP	20 (7 patellar)	NS	Extracorporeal shockwave therapy vs. others
Masiello et al., 2023 [33]	Italy	Blood Transfus.	Tendon and ligament injuries	CRD42021289419	NSP	33 (3 patellar)	NS	PRP vs. control
Matthews et al., 2018 [34]	Australia	Ultrasound Med Biol	Tendinopathy in any location	NS	Males or females of any age, from anyathletic or community back ground	19 (12 patellar)	US	NA
McAuliffe et al., 2016 [35]	Ireland	Br J Sports Med	Achilles and patellar tendinopathy	CRD42015020664	NSP	17 (13 patellar)	Ultrasound	NA
Moraes et al., 2014 [36]	Brazil	Cochrane Database Syst Rev.	Musculoskeletal soft tissue injuries	NS	NSP	19 (1 patellar)	Clinically	PRP vs. placebo, autologous whole blood, dry needling or no platelet-rich therapy
Mousavi et al., 2019 [37]	The Netherlands	Gait Posture	Lower-limb tendinopathy	NS	Distance runners	28 (1 patellar)	NS	NA
Obst et al., 2018 [38]	Australia	Sports Med	Achilles and patellar tendinopathy	NS	NSP	20 (7 patellar)	Clinically	NA
Palazón-Bru et al., 2021 [39]	Spain	Clin J Sport Med	Patellar tendinopathy	NS	NSP	12	Reliability scale	NA
Saltychev et al., 2022 [40]	Finland	Disabil Rehabil	Tendinopathy	NS	Adults (>16 years) with acute or chronic tendinopathy	11 (1 patellar)	NS	Glyceryl trinitrate applied topically vs. placebo, sham, or other treatment
Shim et al., 2023 [41]	UK	Physiotherapy	Tendinopathy	CRD42020168187	Any age or gender	34 (5 patellar)	Clinical	Exercise therapy
Sprague et al., 2018 [42]	USA	Br J Sports Med	Patellar tendinopathy	CRD42016052904	NSP	28	Symptoms isolated to the inferior pole of the patella	NA
Tayfur et al., 2022 [43]	UK	Sports Med	Patellar tendinitis/tenosynovitis/tendinosis	NS	Jumping athletes (any sport)	16	Clinically or ultrasound	NA
Wang et al., 2023 [14]	China	Sports Health	Patellar tendinopathy	NS	Athletes and recruits	11	NS	Prophylactic program vs. control strategy
Woodley et al., 2007 [44]	New Zealand	Br J Sports Med	Achilles tendinopathy and patella tendinopathy and tendinopathy of the common wrist extensor tendon of the lateral elbow	NS	NSP	11 (n patellar NS)	Clinically	Eccentric exercise vs. others

* Comparative treatment studies only. NA: not applicable; NS: not specified; NSP: not specific population.

**Table 2:** AMSTAR-2 scores assessing the quality of the published meta-analyses.

AMSTAR-2\Study	1	2	3	4	5	6	7	8	9	10	11	12	13	14	15	16	Evaluation
Andriolo et al., 2018 [25]	No	No	Yes	Yes	Yes	Yes	Partial Yes	Yes	Yes	No	Yes	No	Yes	Yes	No	No	Critically low-quality
Chen et al., 2019 [26]	No	No	Yes	Yes	Yes	Yes	Partial Yes	Yes	No	No	Yes	No	No	Yes	Yes	No	Critically low-quality
De Bleecker et al., 2020 [27]	Yes	Yes	Yes	Yes	Yes	Yes	Partial Yes	Yes	Yes	No	Yes	Yes	Yes	Yes	No	No	Low-quality
De oliveira Silva et al., 2019 [28]	No	Yes	Yes	Yes	No	No	Partial Yes	Yes	Yes	No	Yes	Yes	Yes	Yes	No	No	Low-quality
Heales et al., 2013 [29]	No	No	Yes	Yes	No	No	Partial Yes	Yes	Yes	No	Yes	Yes	No	Yes	No	No	Critically low-quality
Khan et al., 2023 [30]	No	No	Yes	Yes	Yes	Yes	Partial Yes	Yes	Yes	No	Yes	Yes	No	Yes	Yes	No	Critically low-quality
Kim et al., 2021 [31]	No	Yes	Yes	Yes	Yes	Yes	Partial Yes	Yes	Yes	No	Yes	Yes	Yes	Yes	Yes	No	Moderate-quality
Mani-Babu et al., 2014 [32]	No	No	Yes	Yes	Yes	Yes	Partial Yes	Yes	Yes	No	Yes	Yes	Yes	Yes	No	No	Critically low-quality
Masiello et al., 2023 [33]	No	Yes	Yes	Yes	Yes	Yes	Partial Yes	Yes	Yes	No	Yes	Yes	Yes	Yes	Yes	No	Moderate-quality
Matthews et al., 2018 [34]	Yes	No	Yes	Yes	No	No	Partial Yes	Yes	Yes	No	Yes	Yes	Yes	Yes	Yes	No	Low-quality
McAuliffe et al., 2016 [35]	Yes	Yes	Yes	Yes	Yes	Yes	Partial Yes	Yes	Yes	No	Yes	Yes	Yes	Yes	Yes	No	Moderate-quality
Moraes et al., 2014 [36]	Yes	Yes	Yes	Yes	Yes	Yes	Yes	Yes	Yes	No	Yes	Yes	Yes	Yes	Yes	No	Moderate-quality
Mousavi et al., 2019 [37]	No	No	Yes	Yes	Yes	Yes	Partial Yes	Yes	Yes	No	Yes	Yes	No	Yes	No	No	Critically low-quality
Obst et al., 2018 [38]	No	No	Yes	Yes	Yes	Yes	Partial Yes	Yes	Yes	No	Yes	Yes	Yes	Yes	No	No	Critically low-quality
Palazón-Bru et al., 2021 [39]	Yes	Yes	Yes	Yes	Yes	Yes	Partial Yes	Yes	Yes	No	Yes	Yes	No	Yes	Yes	No	Low-quality
Saltychev et al., 2022 [40]	Yes	No	Yes	Yes	Yes	Yes	Partial Yes	Yes	Yes	No	Yes	Yes	No	Yes	Yes	No	Critically low-quality
Shim et al., 2023 [41]	Yes	Yes	Yes	Yes	Yes	Yes	Partial Yes	Yes	Yes	No	Yes	Yes	No	Yes	Yes	No	Low-quality
Sprague et al., 2018 [42]	No	No	Yes	Yes	Yes	Yes	Partial Yes	Yes	Yes	No	Yes	Yes	Yes	Yes	Yes	No	Low-quality
Tayfur et al., 2022 [43]	No	No	Yes	Yes	Yes	Yes	Partial Yes	Yes	Yes	No	Yes	Yes	No	Yes	No	No	Critically low-quality
Wang et al., 2023 [14]	No	Yes	Yes	Yes	Yes	Yes	Partial Yes	Yes	Yes	No	Yes	Yes	Yes	Yes	Yes	No	Moderate-quality
Woodley et al., 2007 [44]	No	Yes	Yes	No	No	Partial Yes	Yes	Yes	No	Yes	Yes	No	Yes	Yes	No	Yes	Critically low-quality
